# Var2GO: a web-based tool for gene variants selection

**DOI:** 10.1186/s12859-016-1197-0

**Published:** 2016-11-08

**Authors:** Ilaria Granata, Mara Sangiovanni, Francesco Maiorano, Marco Miele, Mario Rosario Guarracino

**Affiliations:** High Performance Computing and Networking Institute, National Research Council of Italy, Via P. Castellino, 111, Napoli, 80131 Italy

**Keywords:** Gene variants, Gene ontology, Annotation, Web-based tool, Next generation sequencing

## Abstract

**Background:**

One of the most challenging issue in the variant calling process is handling the resulting data, and filtering the genes retaining only the ones strictly related to the topic of interest. Several tools permit to gather annotations at different levels of complexity for the detected genes and to group them according to the pathways and/or processes they belong to. However, it might be a time consuming and frustrating task. This is partly due to the size of the file, that might contain many thousands of genes, and to the search of associated variants that requires a gene-by-gene investigation and annotation approach. As a consequence, the initial gene list is often reduced exploiting the knowledge of variants effect, novelty and genotype, with the potential risk of losing meaningful pieces of information.

**Results:**

Here we present Var2GO, a new web-based tool to support the annotation and filtering of variants and genes coming from variant calling of high-throughput sequencing data. Var2GO permits to upload either the unprocessed Variant Calling Format file or a table containing the annotated variants. The raw data undergo a preliminary step of variants annotation, using the SnpEff tool, and are converted to a table format. The table is then uploaded into an on the fly generated database. Genes associated to the variants are automatically annotated with the corresponding Gene Ontology terms covering the three GO domains. Using the web interface it is then possible to filter and extract, from the whole list, genes having annotations in the domain of interest, by simply specifying filtering parameters and one or more keywords. The relevance of this tool is demonstrated on exome sequencing data.

**Conclusions:**

Var2GO is a novel tool that implements a topic-based approach, expressly designed to help biologists in narrowing the search of relevant genes coming from variant calling analysis. Its main purpose is to support non-bioinformaticians in handling and processing raw variant calling data through an intuitive web interface. Furthermore, Var2GO offers a complete pipeline that, starting from the raw VCF file, allows to annotate both variants and associated genes and supports the extraction of relevant biological knowledge.

## Background

Much progress has been made in Next Generation Sequencing (NGS) data analysis. More and more geneticists are introducing this technology in their routine work to exploit a wide, cost-effective approach in identifying and studying genetic variants across the genome. Whole-genome and whole-exome sequencing are widely used to investigate these variants as cause of rare and complex diseases. Handling genome or exome data is a powerful opportunity but not poor in challenges, due to technical difficulties in managing such big amounts of data. Transforming raw sequence reads into meaningful information might be a tough task, especially for small labs. Although exome sequencing reports much less variants compared to genome sequencing [1], the output coming from the entire pipeline of variant calling is a huge VCF (Variant Calling Format) file containing hundreds of thousand rows. The VCF file contains both single-nucleotide polymorphisms (SNPs) and small insertions and deletions (INDELs), annotated using several variants identification tools, such as ANNOVAR [2], SnpEff [3], AnnTools [4]. These software provide several information such as the affected genes, the effects of the variants at the level of the protein products, or the minor allele frequency. However, they do not support any further investigation aimed to identify genes significant to the topic of study. To give a biological meaning to the obtained data, the scientist needs to filter the relevant information from hundreds of thousands of SNPs and INDELs called, since only a subgroup of variants is pertinent to the given domain of interest [5, 6].

A large amount of scientific literature proves that the Gene Ontology (GO), from the Gene Ontology Project [7], is an invaluable source of the most sophisticated meanings of biological systems available to scientists [8]. GO annotations provide a bridge between the gene product and its function, its cellular localization and the biological processes in which it is involved. This knowledge is organized into three different GO ontologies: *molecular function*, *cellular component* and *biological process*, respectively. We focused on the GO annotation as a structured, precisely defined, common, controlled vocabulary for describing the roles of genes and gene products in any organism [9].

Many tools have been developed to annotate lists of genes in terms of the biological role of their products, and to filter the huge VCF variants lists. However, they often require advanced computational skills for both the installation process and their usage [10, 11]. When a web service is available, as in [12], usually only a list of genes is given in output, thus requiring further processing to match the initial data with the list of new annotations. This is an essential step to retain all the information collected during the pre-processing steps (quality parameters, position, variant ID, sample genotype and others), since they might be used to better investigate among variant features and obtain more reliable results.

As a consequence, the initial variant list is often greatly reduced by applying simple filtering criteria such as variants effect prediction, novelty and genotype. While this approach might help in reducing the size of the data, it greatly increase the risk of losing meaningful pieces of information.

Examples of available tools for gene annotation include: GoGene [12], which is conceived to allow gene annotation through GO and MeSH (Medical Subject Headings) vocabularies. It requires a list containing gene names/identifiers as input. The output list with annotation is easily viewable by the web interface, but, although the links to export the list in different file formats are provided, they do not seem to have any content. Furthermore, it does not work with input list containing more than 1000 genes; AnnoKey [13], which is usable exclusively from command line and it has a gene number limit in online mode; SNP2GO [14], which is an R package, hence requiring the user to have programming skills. Moreover, it is not available for all the recent R versions (tested under R v3.2.0 and v3.2.1).

Other software are conceived to allow the direct interaction with the variant table to apply filtering strategies: GEMINI [15], KGGSEQ [16], Plink/SEQ [17], which are more focused on variant rather than gene annotation, and all exclusively usable from command line. One available tool for visualizing annotated sequence variants and interacting with the VCF input file is SVA [18], which requires high-performance workstations or servers to properly work. Other available software include VCF-Miner [10] and EVA [11], but both of them need the installation of a complete web stack as a prerequisite. The most user-friendly tool is VarSifter [19], a Java-based software which requires a minimal installation. However, it can be mainly used as a filtering tool, since it does not offer further annotation features.

To support the researchers in the process of annotating and filtering all the variants obtained from sequencing experiments, we have developed a web-based tool named *Var2Go*. To the best of our knowledge, Var2GO is the first web tool which permits to upload a complete raw variants file, to annotate both the variants and the related genes, and filter them in an interactive way, obtaining a reduced file with all the needed information. Var2Go accepts as input either a raw VCF file or a tab-delimited annotated table with a maximum size of 500 MB. In the first case, the user must select the reference genome for the variant annotation (at the moment the available genome assemblies are hg19 and hg38 for *Homo Sapiens*, mm9 and mm10 for *Mus musculus*). The VCF file is annotated using the SnpEff 4.2 [3] which produces a new VCF file in a format compliant with the latest annotation standard. After, the resulting file is converted in table format using SnpSift [20]. SnpEff annotates more than one transcript for each gene variant. We decided to keep and show the most relevant one, based on the selection strategy described in the software documentation available at the SnpEff website [21]. Instead, when uploading a file in table format, the user must select the species of interest and the fields containing the essential information (i.e. Gene symbol, Chromosome, Position, Reference and Alternative allele). Var2GO extracts the annotations for each gene among the three GO ontologies, and creates on the fly a relational database in which all the fields of the table and the relative content are stored. Through the web interface, it is possible to specify a list of key terms (whole words or portions) considered by the user as pertinent to the specific topic of study. Furthermore, Var2GO permits to dynamically interact with the input table applying filters to the information contained into the columns. Lastly, it is possible to select columns to be included in the resulting output file.

## Implementation

The Var2GO web interface is implemented using the PHP language on an Apache webserver. Javascript and JQuery are used for client side programming. The Plupload 2.1.8 plugin [22] is used to manage chunked transfers of large files. The input file pre-processing and the on the fly database building steps are implemented using the Perl language and a MySQL DBMS (Data Base Managment System). Variants annotation are obtained through SnpEff 4.2 [3]. Table conversion is performed using SnpSift [20]. Annotation from GO are fetched using the online MySQL service provided by the GO consortium. The main page of Var2GO website allows the user to select the file to upload. The input file must be either a raw VCF or a tab-delimited table file. The user will be asked to specify some details on the uploaded file. For the raw VCF the assembly version should be provided. For the table file, the user must choose the species among the common ones used by GO. Moreover, the user must select which column headers contain relevant data: the chromosome id, the starting position of the variant, the reference sequence, the alternative sequence, and the gene name. These information are mandatory and essential for the correctness of the subsequent elaboration.

It is also possible to choose whether to convert the GT (Genotype) characters into HOM/HET/HOMREF nomenclature by a pre-processing step. After the database creation, the user can visualize the loaded data and specify the keywords and filters to use, using dropdown lists. The query is built using the specified filtering criteria on the columns content and keywords connected by selecting boolean operators.

After the annotation and filtering processes are complete, the user can download the resulting table in a tab-delimited format. If the variants annotation step is requested, two gzip files, one for the complete table and one for the relative transcripts info, are available for downloading. Furthermore, it is possible to download the entire database created by Var2Go. This option has been thought for users who have more advanced computational skills and can store the information ready to be filtered and parsed.

## Results and discussion

**Fig. 1:**
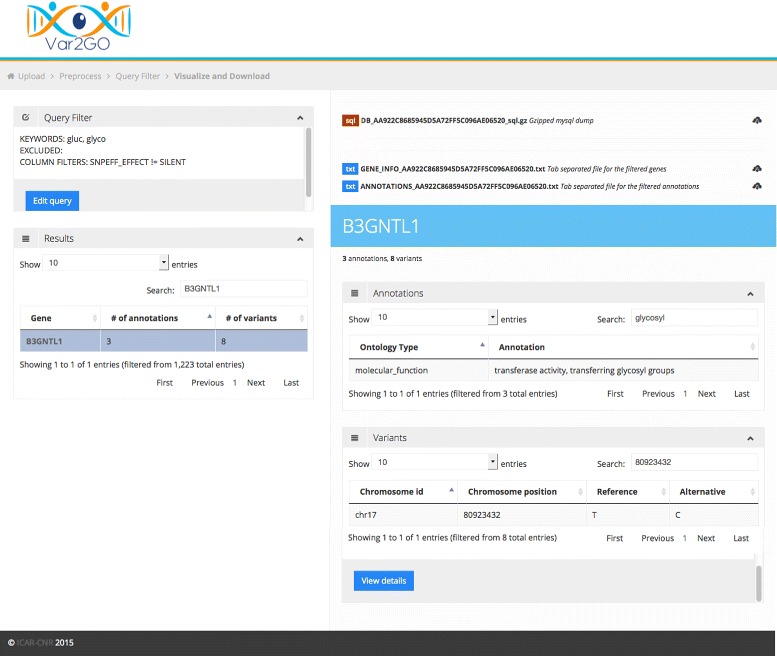
Screenshot of the result page of Var2GO webapp. **Top left:** "Query Filter" area. The user defined filtering criteria are shown. **Bottom left:** "Results" area. The genes satisfying the filtering criteria are shown. The name of the gene symbol, the number of associated variants and GO annotations are reported. The total number of retrieved genes is given at the bottom of the table ("entries"). **Bottom right:** gene details area. When a gene is selected from the "Results" area, its details appear here. Two tables are shown, one concerning the GO annotation and the other one containing some basic information on each associated variant. Clicking the "View details" button a new page will show the whole detailed information. **Top right:** download area. The downloadable files are shown

Var2GO has a simple and user-friendly interface (see Fig. 1). The users is guided through the few steps needed to upload and create the database. After, the choice of the filters and key terms can be performed using a very intuitive approach. Results can be downloaded either as a simple tab-separated file or as a complete database, for more experienced users. The usefulness of Var2GO is tested on a data set coming from an exome sequencing experiment.

### Case study: Glycogen storage disease exome sequencing

We used an exome sequencing dataset coming from a variant discovery study on a family with polyglucosan body myopathy [23]. Polyglucosan body myopathies (PGBMs) are a group of glycogen storage diseases (GSDs) which affects almost exclusively the striated muscle [24]. The glycogen storage disease are rare inherited metabolic disorders caused by malfunction of enzymes involved in synthesis or degradation of glycogen [25]. There are over 12 different types classified accordingly to the involved enzyme and affected tissue. Since exogenous glucose is metabolized and stored in the liver and skeletal muscle, these are the two most involved tissues [26]. The dataset we used was composed of samples coming from three affected relatives (2 siblings, 1 cousin) who presented a late-onset PGBM. The VCF file was obtained through a variant calling pipeline performed by the authors. SnpEff [3] and ANNOVAR [2] tools were used to annotate each called variant in terms of amino acid change, gene name, functional class, impact, frequency in NHLBI Exome Variant Server (ESP6500) [27] and 1000 Genomes Project [28, 29], and causative effects by the SIFT prediction algorithm [30]. The annotated VCF file was then converted to a tab-delimited format by VariantsToTable, a walker of the Genome Analysis ToolKit (GATK) [31]. The table, composed by 57 columns reporting variant information obtained from both calling and annotation steps, and 160501 variant rows, was uploaded to Var2GO. We filtered the variants by several annotations, such as ESP 6500 allele frequency, VQSLOD filter (derived by variant quality score recalibration walker of GATK to assign a well-calibrated probability to each variant call), SnpEff functional class, genotype and depth of coverage. In detail, we asked Var2GO to retain not silent variants which passed the VQSLOD filter, had a ESP 6500 minor allele frequency smaller than 0.02, a depth of coverage per sample greater than 20, and were in common to the three samples. Applying these filtering criteria we obtained a table containing 8978 genes, less than 50 % of the initial list (19453 genes). In order to narrow the search to the genes which had functionality related to the topic of interest, we applied a filter to the GO annotation field by typing the following keywords: ‘gluc’ (for ‘glucose’, ‘glucan’, ‘glucotransferase’, etc.), and ‘glyco’ (for ‘glycogen’, ‘glycolitic’, ‘glicosylation’, etc.). The resulting table contained 949 genes, each with more than one variant called and multiple GO annotations. Then, we applied a filter also to the field of the 1000 Genome allele frequency (<0.02) and we obtained 352 genes. At this point, the reduced table was ready to be downloaded and easily handled and interpreted. The reduced size of the resulting gene list allowed to retrieve additional information from pathway and/or gene-disease association studies, using, for example, the Database for Annotation, Visualization and Integrated Discovery (DAVID) [32]. The submission of our list to this resource and the selection of KEGG [33], BioCarta [34], Reactome [35], OMIM [36] annotation categories, resulted in 44 chart records ranked in according to the *p*-value. The most significant term was ‘*REACT_474:Metabolism of carbohydrates*’ from Reactome, enriched by 20 genes and with a fold enrichment score of 6.0248. At this point, we better investigated these 20 genes and we found that 4 of them are associated to glycogen storage disease as reported by the Human Phenotype Ontology project (HPO) [37, 38], namely LDHA, PHKG2, ENO3 and GYG1. Hence, the use of Var2GO allowed to easily find the GYG1 variant, confirming the result obtained by Fanin et al. [23]. Handling a reduced list of genes permits to better exploit the available knowledge and the online resources, thus to deeply investigate the variant genes without losing precious information. Moreover, besides known disease genes, Var2Go allows to explore variants in genes which may be involved in the pathophysiological process.

## Conclusions

We developed Var2Go to support researchers in handling and interpreting gene variant data obtained from high-throughput sequencing experiments. Thanks to an user-friendly and web-based interface, this tool allows to annotate, filter and search large variant files with a small effort from the user and no programming skills or computational resource required, except a modern browser. Few easy steps are needed to reduce the initial table to variants having the desired quality and features, and to genes having ontology terms related to the domain of interest. We are currently working to add the HPO annotation for human genes, and more genomic databases to allow the variants annotation of different species. Further, we are planning to provide Var2Go as a cloud app in order to improve performance and allow the processing of larger files.

## Availability and requirements

**Project name**:Var2GO;**Project home page**: http://www-labgtp.na.icar.cnr.it/VAR2GO **Operating system(s):** Platform independent.**Programming languages:** Perl, PhP, Java-script, JQuery, css, HTML5;**Other requirements:** A Javascript-enabled browser is required;**License:** GPLv3;**Any restrictions to use by non-academics**: no.

## Abbreviations

NGS: Next generation sequencing
VCF: Variant calling format
SNPs: Single nucleotide polymorphism(s)
INDELs: insertion(s) and deletion(s)
GO: Gene ontology
DBMS: Data base management system
GT: GenoType
HOM/HET/HOMREF: HOMozygous/HETerozygous/HOMozygous REFerence
PGBMs: PolyGlucosan body myopathies
GSDs: glycogen storage diseases
LDHA: Lactate dehydrogenase A
PHKG2: PHosphorylase kinase gamma 2
ENO3: Enolase 3
GYG1: Glycogenin 1
